# A prognostic model for use before elective surgery to estimate the risk of postoperative pulmonary complications (GSU-Pulmonary Score): a development and validation study in three international cohorts

**DOI:** 10.1016/S2589-7500(24)00065-7

**Published:** 2024-07

**Authors:** NIHR Global Health Research Unit on Global Surgery, STARSurg Collaborative

## Abstract

**Background:**

Pulmonary complications are the most common cause of death after surgery. This study aimed to derive and externally validate a novel prognostic model that can be used before elective surgery to estimate the risk of postoperative pulmonary complications and to support resource allocation and prioritisation during pandemic recovery.

**Methods:**

Data from an international, prospective cohort study were used to develop a novel prognostic risk model for pulmonary complications after elective surgery in adult patients (aged ≥18 years) across all operation and disease types. The primary outcome measure was postoperative pulmonary complications at 30 days after surgery, which was a composite of pneumonia, acute respiratory distress syndrome, and unexpected mechanical ventilation. Model development with candidate predictor variables was done in the GlobalSurg-CovidSurg Week dataset (global; October, 2020). Two structured machine learning techniques were explored (XGBoost and the least absolute shrinkage and selection operator [LASSO]), and the model with the best performance (GSU-Pulmonary Score) underwent internal validation using bootstrap resampling. The discrimination and calibration of the score were externally validated in two further prospective cohorts: CovidSurg-Cancer (worldwide; February to August, 2020, during the COVID-19 pandemic) and RECON (UK and Australasia; January to October, 2019, before the COVID-19 pandemic). The model was deployed as an online web application. The GlobalSurg-CovidSurg Week and CovidSurg-Cancer studies were registered with ClinicalTrials.gov, NCT04509986 and NCT04384926.

**Findings:**

Prognostic models were developed from 13 candidate predictor variables in data from 86 231 patients (1158 hospitals in 114 countries). External validation included 30 492 patients from CovidSurg-Cancer (726 hospitals in 75 countries) and 6789 from RECON (150 hospitals in three countries). The overall rates of pulmonary complications were 2·0% in derivation data, and 3·9% (CovidSurg-Cancer) and 4·7% (RECON) in the validation datasets. Penalised regression using LASSO had similar discrimination to XGBoost (area under the receiver operating curve [AUROC] 0·786, 95% CI 0·774–0·798 *vs* 0·785, 0·772–0·797), was more explainable, and required fewer covariables. The final GSU-Pulmonary Score included ten predictor variables and showed good discrimination and calibration upon internal validation (AUROC 0·773, 95% CI 0·751–0·795; Brier score 0·020, calibration in the large [CITL] 0·034, slope 0·954). The model performance was acceptable on external validation in CovidSurg-Cancer (AUROC 0·746, 95% CI 0·733–0·760; Brier score 0·036, CITL 0·109, slope 1·056), but with some miscalibration in RECON data (AUROC 0·716, 95% CI 0·689–0·744; Brier score 0·045, CITL 1·040, slope 1·009).

**Interpretation:**

This novel prognostic risk score uses simple predictor variables available at the time of a decision for elective surgery that can accurately stratify patients’ risk of postoperative pulmonary complications, including during SARS-CoV-2 outbreaks. It could inform surgical consent, resource allocation, and hospital-level prioritisation as elective surgery is upscaled to address global backlogs.

**Funding:**

National Institute for Health Research.

## Introduction

Pulmonary complications are a common and lethal sequela of elective surgery, affecting 2–62% of patients depending on the operative approach and casemix.^1^ As elective surgery is upscaled to address a growing global backlog from the COVID-19 pandemic, pulmonary events pose a major threat to patient safety.^2,3^ Such events were implicated in 37% of postoperative deaths before the pandemic, and as many as 66% of deaths during the pandemic due to additional risks of perioperative SARS-CoV-2 infection.^4^ Pulmonary complications of surgery have a major effect on hospital resource use and demand on critical care services, which remain under pressure during COVID-19 pandemic recovery in many parts of the world.^5^

Several prognostic risk models for postoperative pulmonary complications have been reported in the literature. However, in a 2022 systematic review and external validation study,^6^ few existing models had been externally validated and none showed acceptable performance (lower 95% CI estimate of the area under the receiver operating curve [AUROC] test characteristic ≥0·7). All risk scores that reported external validation used at least one complex variable that might not be available in the outpatient clinic at the time of decision for surgery or in resource-limited environments, and were at a high or unclear risk of bias. Methods for exploring interactions between candidate risk variables using structured machine learning have not been used previously in the development of risk scores for estimating risk of pulmonary complications. No existing scores reflect additional risk to patients during local outbreaks of SARS-CoV-2 for patients undergoing surgery.^7^

Identification of patients at high risk of pulmonary complications after elective surgery is likely to have several benefits during ongoing pandemic recovery efforts. First, it could be used to inform patient consent for surgery. Second, it could be used to inform a decision to delay elective surgery until a time when community SARS-CoV-2 infection rates are lower or vaccination (or booster doses) are provided.^8^ Third, it would help to stratify the use of resource-intensive interventions to modify risk and improve capacity to rescue patients from complications.^9^ Finally, it could inform case prioritisation to address the growing backlog of cancelled elective surgeries worldwide.^10^

This study aimed to derive and validate a novel prognostic model to estimate risk of pulmonary complications of elective surgery using data from three large, international, prospective cohort studies. The secondary aim was to compare a model developed using an advanced structured machine learning approach with a simpler penalised regression-based approach.

## Methods

### Study design

This prognostic model derivation and validation study used data from three international, prospective, multicentre cohort studies to develop a tool to estimate the risk of postoperative pulmonary complications for adult patients undergoing planned (elective) surgery. The derivation dataset was from the GlobalSurg-CovidSurg Week study, which aimed to determine the optimal timing for surgery following SARS-CoV-2 infection. A full description of methods and findings of this study has been published.^8^ This study dataset was selected for model derivation as it was the largest and most data-rich for model fitting, with the broadest inclusion criteria (ie, most generalisable). The protocol of GlobalSurg-CovidSurg Week was prospectively registered at ClinicalTrials.gov (NCT04509986). The model developed in this cohort was then externally validated in two independent datasets. The first was from CovidSurg-Cancer, a study of adult patients with a surgically curable cancer who underwent surgery during the COVID-19 pandemic.^2^ The study was done in accordance with a preregistered protocol (NCT04384926). The second validation dataset was from RECON, a study of pulmonary complications after major abdominal surgery.^4^

All three studies collected routine, anonymised data with no change to clinical care pathways, and secured approvals for participating hospitals in line with local and national regulations before entering data into the study. Informed patient consent was obtained if this was necessary to comply with local or national regulations. Country-specific ethics or relevant clinical governance approvals are available upon request. Collaborators were required to confirm evidence of relevant approvals before entering patient data in all three included studies. This approvals process was delegated to the site principal investigators at each participating centre, and one or more national leads in each participating country (appendix pp 33–213).

The study authors collaborated across the leadership teams for all three cohort studies and had direct access to the original datasets. This study is reported with reference to the transparent reporting of a multivariable prediction model for individual prognosis or diagnosis (TRIPOD) guidelines.^11^ Two independent statisticians assessed risk of bias in the study using the prediction model risk of bias assessment tool (PROBAST).^12^

### Participants

GlobalSurg-CovidSurg Week^8^ included all consecutive patients undergoing surgery between Oct 5 and Nov 1, 2020. Any centre worldwide performing inpatient surgery was eligible for inclusion. Participating centres could include children and adults from one or more specialty groups, so long as consecutive sampling was done. This included both elective and emergency surgery, and patients undergoing all operation types for any condition.

CovidSurg-Cancer^2^ included consecutive adult patients with a surgically curable cancer who underwent surgery from the emergence of COVID-19 up to Aug 31, 2020. Any hospital worldwide that performed elective cancer surgery in an area affected by the COVID-19 pandemic was eligible. Data from the 15 most common operated, solid tumour types were collected with colleagues collaborating across ten surgical oncology disciplines. Patients who had clinical symptoms consistent with COVID-19 or who were confirmed to have SARS-CoV-2 infection (by quantitative RT-PCR or thoracic CT imaging, or both) at the time of surgery were excluded.

RECON^4^ included consecutive adult patients undergoing a broad range of elective major abdominal surgeries, including abdominal visceral resection, reversal of stoma, open vascular surgery, anterior abdominal wall hernia repair, or transplant surgery, using any operative approach. Planned day-case procedures and abdominal surgeries without visceral resection were excluded. Eligible patients were identified at each participating hospital during two data collection periods: Jan 1 to April 30, 2019 (in the UK and Ireland), and Sept 1 to Oct 31, 2019 (in Australia).

This derivation and validation study included only adult patients (aged ≥18 years) undergoing planned (elective) surgery. Elective surgery was defined as any operation for which the decision for surgery was made before the hospital admission during which the operation took place. No restrictions on operation type, approach, or indication for surgery were imposed. Patients with suspected or confirmed SARS-CoV-2 infection at the time of surgery (ie, a positive nasopharyngeal swab with RT-PCR test confirming the presence of SARS-CoV-2, or strongly indicative signs or symptoms when testing facilities were not available) were excluded. Patients undergoing local excision or endoluminal surgery were excluded.

### Data governance and data quality

Data were collected online and stored on a secure server running the Research Electronic Data Capture (REDCap, Vanderbilt University, Nashville, TN, USA) web application,^13^ based in the University of Birmingham, UK. Data quality rules were built into the dataset, and study definitions were provided inline with data entry to improve reproducibility. Patients were pseudonymised at the point of entry using a unique REDCap identifier, with linked patient identifiers stored securely by the participating site. Data quality was checked continuously, with data clarification requests sent to sites at twice monthly intervals during the study period, and during data cleaning, in which inconsistencies or missing data were identified. Previous international outcomes studies from our groups have shown that this collaborative methodology has greater than 95% case ascertainment and greater than 98% data accuracy.^14^ In these three studies, if a specialty within a hospital was unable to confirm consecutive enrolment, their specialty’s data were excluded from analysis.

### Outcomes

The primary outcome was postoperative pulmonary complications (PPC) within 30 days of surgery (with day 0 as the day of surgery). PPC was defined as a composite of pneumonia, acute respiratory distress syndrome, and unplanned use of invasive or non-invasive mechanical ventilation. This was adopted for two reasons: to mirror this outcome definition in several major randomised trials and cohort studies,^15^ and to include outcomes plausibly linked to SARS-CoV-2 infection with relevance to practice during pandemic recovery.^5^ The 30-day PPC rate was the predefined primary outcome in the GlobalSurg-CovidSurg Week and CovidSurg-Cancer cohort study protocols and the key clinical outcome in the RECON study. Outcome assessment was done on or as close as possible after the 30th postoperative day by a trained member of the local clinical team. As front-line clinical investigators were aware of patients’ relevant medical history, masking of outcome assessment to some predictor variables was not feasible. Diagnosis was, however, supported using radiological assessments, reported locally by a radiologist masked to several of the predictor variables. Full definitions are included in the appendix (pp 16–21). The secondary outcome measure was the 30-day postoperative mortality rate (all-cause).

### Predictor variables

Candidate predictor variables (n=53) were identified in a systematic review of existing risk scores and then refined with multidisciplinary clinical input, gaining consensus from surgery, anaesthesia, and critical care experts. To improve the adoption of the risk stratification tool, we planned to include only predictor variables that were universally available without the need for additional tests, and that were relevant to low-resource countries and hospitals.^6^ We selected variables that are routinely available to clinicians at the point of a decision for elective surgery (eg, preoperatively in an outpatient clinic or inpatient ward) to inform preoperative decision making and consent; we did not include intraoperative variables as they cannot influence clinical pathways determined preoperatively, or physiological parameters as they are challenging to collect reliably in diverse, multicountry settings. Therefore, 13 candidate patient-level predictors were selected a priori to be included in model development (appendix pp 16–21): (1) patient factors: age in years (collected in primary data as a categorical variable: 18–29, 30–39, 40–49, 50–59, 60–69, 70–79, 80–89, and ≥90), sex (female or male), American Society of Anesthesiologists (ASA) grade (1, 2, 3, or ≥4), Revised Cardiac Risk Index score (0, 1, 2, or ≥3), pre-existing respiratory disease (yes or no), and smoking status (non-smoker, stopped smoking >6 weeks before surgery, or current smoker); (2) disease factors: compartment (thoracic, abdominopelvic, head and neck, limb, or other), indication (benign or cancer), and pre operative SARS-CoV-2 test (not-performed or negative); (3) operation factors: anaesthetic type (general, regional, or local) and operation grade (minor or major, defined according to the BUPA operation severity classification system listed in the schedule of procedures); (4) location factors: hospital type (COVID-19-free surgical pathway or no defined pathway), country income (high-income country or HIC, upper-middle-income country or UMIC, or low-middle-income country or LMIC [including both lower-middle and low-income countries], according to the World Bank classification), and community SARS-CoV-2 risk (low or high, based on the 14-day cumulative case notification rate in the area of the hospital around the date of surgery).^16^

Country income has a known association with risk of perioperative complications, such as surgical site infection, anastomotic leak, and mortality;^14^ the reasons for this are multifactorial but are considered to reflect a hospital’s or health system’s capacity to rescue. This describes the ability of hospitals to identify, intervene, and rescue patients from complications, requiring infrastructure, consumables, facilities, training, staffing, and contextually relevant protocols.^17^ Patients with recent or active preoperative SARS-CoV-2 infection were excluded. As we could not predict potential risk of postoperative SARS-CoV-2 infection at an individual patient level using only information available preoperatively, we used community SARS-CoV-2 rates as a proxy for a patient’s risk of nosocomial (ie, in-hospital from patients being treated for COVID-19) or community (ie, after discharge while recovering at home) transmission. Due to differences in detection of SARS-CoV-2 with the availability of facilities for testing and notification across income settings, we preplanned to handle the interaction between these two location factors by creating a stratified factor of community SARS-CoV-2 risk by income.^18,19^

Predictor variables were recorded at the time of patient entry (ie, day of surgery or day 0). Where one of the candidate variables was not collected in a dataset, and its value was implicit from the study protocol, it was imputed (ie, preoperative SARS-CoV-2 test imputed as not-performed, and community SARS-CoV-2 risk imputed as low in the pre-pandemic RECON dataset).

A complete-case analysis was preplanned if missing data were both missing at random and in a low number of samples (<5%).^20^ If both assumptions were not met, we planned to impute missing predictor variable data using multiple imputation by chained equations. The number of patients with missing data was reported for each predictor variable across each of the three studies.^21^

### Model derivation

First, we assessed the adequacy of our sample size for use in new model development,^22^ using an estimate of the predicted AUROC (0·700) and prevalence (3·0%) from a contemporary systematic review.^6^ We estimated a minimum sample size requirement of 21 299 patients, and 639 events with RStudio using finalfit, tidyverse, cvAUC, tidymodels, workflowsets, finetune, pROC, rms, glmnet, dcurves, and shiny packages.

Two models were trained using the derivation data during model comparison. The first model consisted of a penalised logistic regression model, the least absolute shrinkage and selection operator (LASSO), in which logistic regression with an extra penalty term shrinks covariate coefficients towards zero, allowing the generation of sparse models and concurrently performing feature selection.^23^ The second model to be applied was gradient boosting decision trees (ie, XGBoost), which is commonly recognised as a best-in-class machine learning algorithm for datasets of moderate size, particularly where significant class imbalance exists.^24^ XGBoost can acknowledge more complex interactions and so is hypothesised to obtain better predictive capabilities.^25^ However, because of its complexity, the ability to explain and understand predictive reasoning is hindered (black box machine learning).^26^ The derivation set was split into a random 75% (training) and 25% (testing), and both models were fit in the training set through 10-times cross-validation and hyperparameter tuning using tune race analysis of variance with grid search. The hyperparameters tuned were lambda for LASSO and learning rate, maximum tree depth, loss reduction, and minimum number, with trees set to 1000 for XGBoost.

During the model selection phase, model performance and feature importance were compared between the two models. Performance was estimated using the model discrimination, which describes models’ ability to rank patients based on risk. Discrimination was evaluated using the AUROC. Confidence intervals were assessed from the cross-validated models using the cvAUC package of R version 1.1.4. Variable importance was assessed for both modelling approaches to better understand the model’s decision making.^27^ Final model selection was balanced between (in order of priority): (1) performance; (2) interpretation and explainability; (3) the number of predictor variables (ie, fewer predictor variables assumed to be easier to adopt in clinical practice). If the LASSO model was selected, we preplanned to refit this model using resampling (2000 bootstraps) and average the coefficients across all resamples.^28^ To generate a pragmatic prognostic index that could be used at a patient’s bedside, we planned to scale and round the averaged penalised regression coefficients (eg, mean coefficients multiplied by 3 and rounded).^25^ If the XGBoost model was selected, we planned to conduct further bootstrapping and feature importance analysis (eg, DALEX).^29^

The performance of the final model (hereafter the GSU-Pulmonary Score) was assessed using the testing split previously derived (internal validation) and both discrimination and calibration were measured. Discrimination was assessed with 95% CIs calculated by bootstrap resampling (2000 samples). Calibration, measuring the agreement between predicted and observed risk, was examined through the Brier score, calibration in the large (CITL), and calibration slope, and visualised through reliability diagrams across risk deciles.

### Model validation

After internal validation, the GSU-Pulmonary Score was externally validated using two large, international, prospective datasets described above (CovidSurg-Cancer and RECON). We assessed the GSU-Pulmonary Score’s performance (discrimination and calibration) in the external datasets, overall and in three preplanned subgroups deemed to be of high clinical importance: (1) major versus minor operation grade; (2) low (ASA grade 1–2) versus high (ASA grade ≥3) risk; and (3) high-income versus low-income and middle-income countries (including upper-middle-income, lower-middle-income, and low-income countries). We also did sensitivity analyses to address death as a competing risk, excluding patients who did not have a PPC diagnosis and died before their 30-day primary outcome assessment. To aid in the interpretation of the external validation, we explored whether reproducibility or transportability was being evaluated.^30^ In brief, this analysis has three phases. First, assessing the relatedness of datasets. Second, comparing model discrimination and calibration in the derivation and validation set. Third, interpretation of external validation results. Finally, we performed decision curve analysis.^31^ This allowed us to make a clinical judgement about the relative value of benefits (treating a true positive) and harms (treating a false positive) associated with application of the score. The standardised net benefit was plotted against the threshold probability in both validation datasets.

All analysis was done in R Foundation for Statistical Computing V4.02 (Vienna, Austria) and RStudio using finalfit, tidyverse, cvAUC, tidymodels, workflowsets, finetune, pROC, rms, glmnet, dcurves, and Shiny packages. To ensure transparency in research, all scripts and code used have been uploaded to GitHub.

### Patient and public involvement

A patient advisory group was formed with Patients and Research Together at Bowel Research UK (registration number 1120460) to provide active input into both the GlobalSurg-CovidSurg Week and CovidSurg-Cancer studies. This group helped to prioritise this research question, provide important input on the protocol, and coproduce patient-facing materials to disseminate research findings. Our methodology for rapid, responsive patient involvement during COVID-19 has been published,^32^ and our updated patient-facing resources are open access and available online. All patient partners are included as equal coauthors in our collaborative author group.

### Role of the funding source

The funder of the study had no role in study design, data collection, data analysis, data interpretation, or writing of the report.

## Results

**Figure 1:**
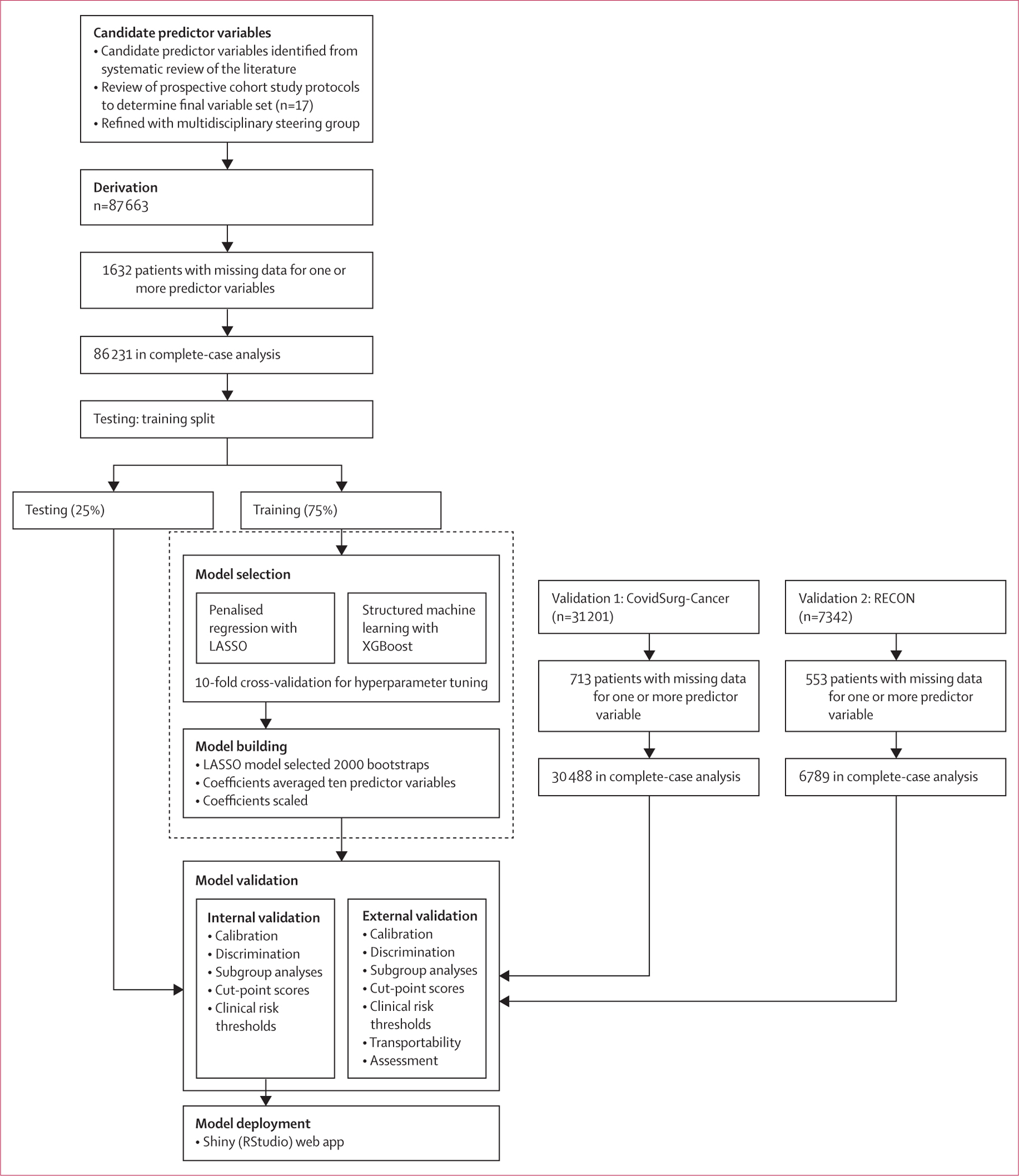
Study flowchart LASSO=least absolute shrinkage and selection operator.

Data from 126 410 patients undergoing surgery in 119 countries were eligible (figure 1). This included 87 863 patients in 1158 hospitals from 114 countries in the derivation dataset, 31 205 patients in 726 hospitals from 75 countries in the CovidSurg-Cancer validation dataset, and 7342 patients in 150 hospitals from three countries in the RECON validation dataset. The overall rate of missing data was very low (<0·1% overall and <2% for all candidate predictor variables; appendix p 2) and appeared to be missing at random (appendix p 11), so the preplanned complete-case analysis was done. The complete-case analysis included 86 231 patients in model development, and 30 492 from CovidSurg-Cancer and 6789 from RECON in external validation.

Table 1 presents differences in casemix and outcomes across the three cohorts. In the derivation data, 2·1% (95% CI 2·0–2·2; 1781/86 231) of patients had a pulmonary complication. The 30-day PPC rate was higher in both the CovidSurg-Cancer (3·9%, 95% CI 3·7–4·1; 1185/30 492) and RECON (4·7%, 95% CI 4·2–5·2; 318/6789) validation studies. The postoperative mortality rate was 0·7% (0·6–0·8; 601/86231, three missing) in the derivation data, 1·1% (95% CI 0·9–1·2; 320/30492, 24 missing) in CovidSurg-Cancer, and 0·4% (95% CI 0·3–0·6; 27/6789, 223 missing) in RECON.

**Figure 2:**
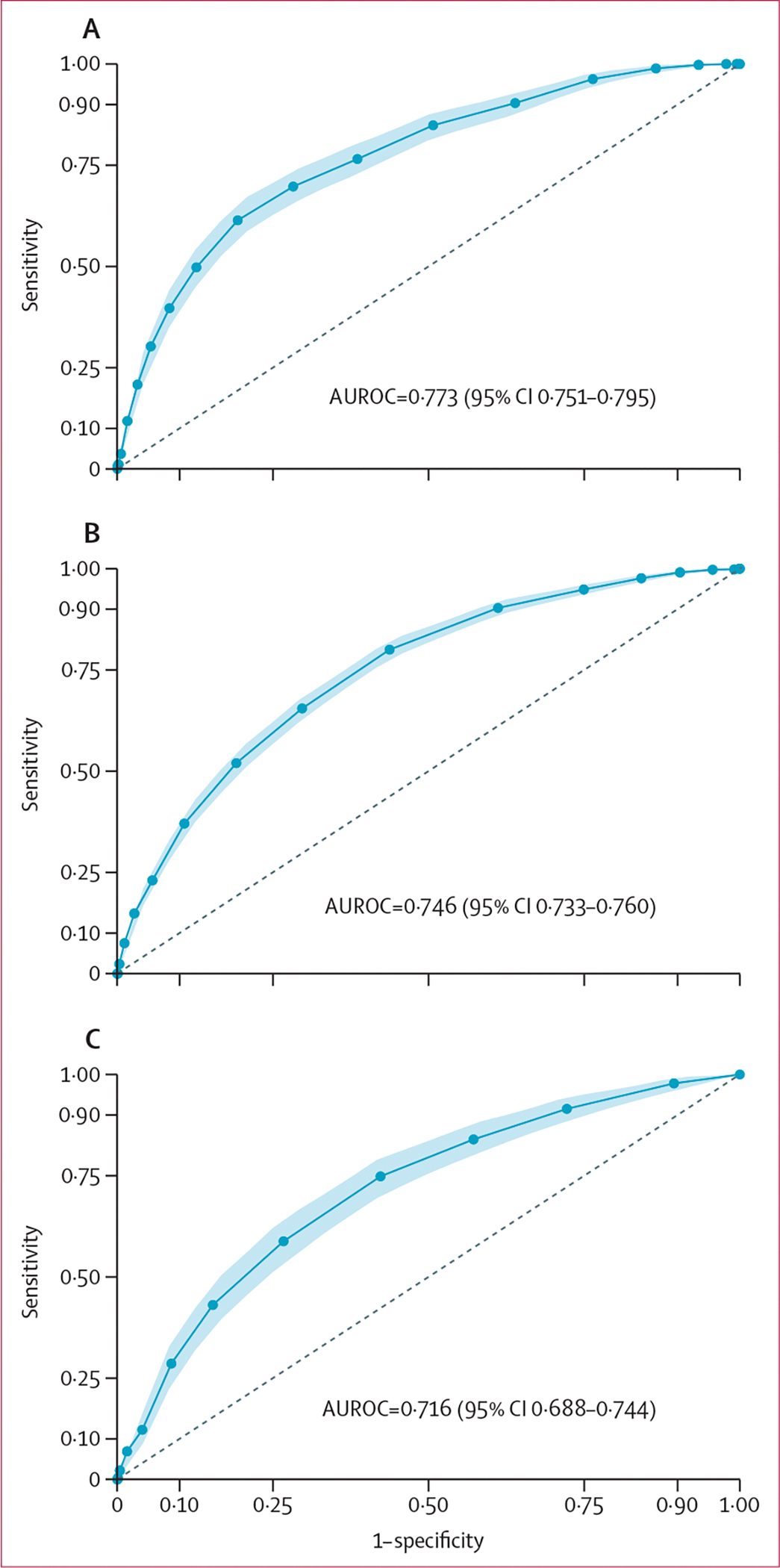
Discrimination of GSU-Pulmonary Score in derivation (A) Testing dataset (GlobalSurg-CovidSurg Week). (B) CovidSurg-Cancer validation dataset. (C) RECON validation dataset. 95% CIs were derived from 2000 bootstrap resamplings. AUROC=area under the receiver operating curve.

**Figure 3:**
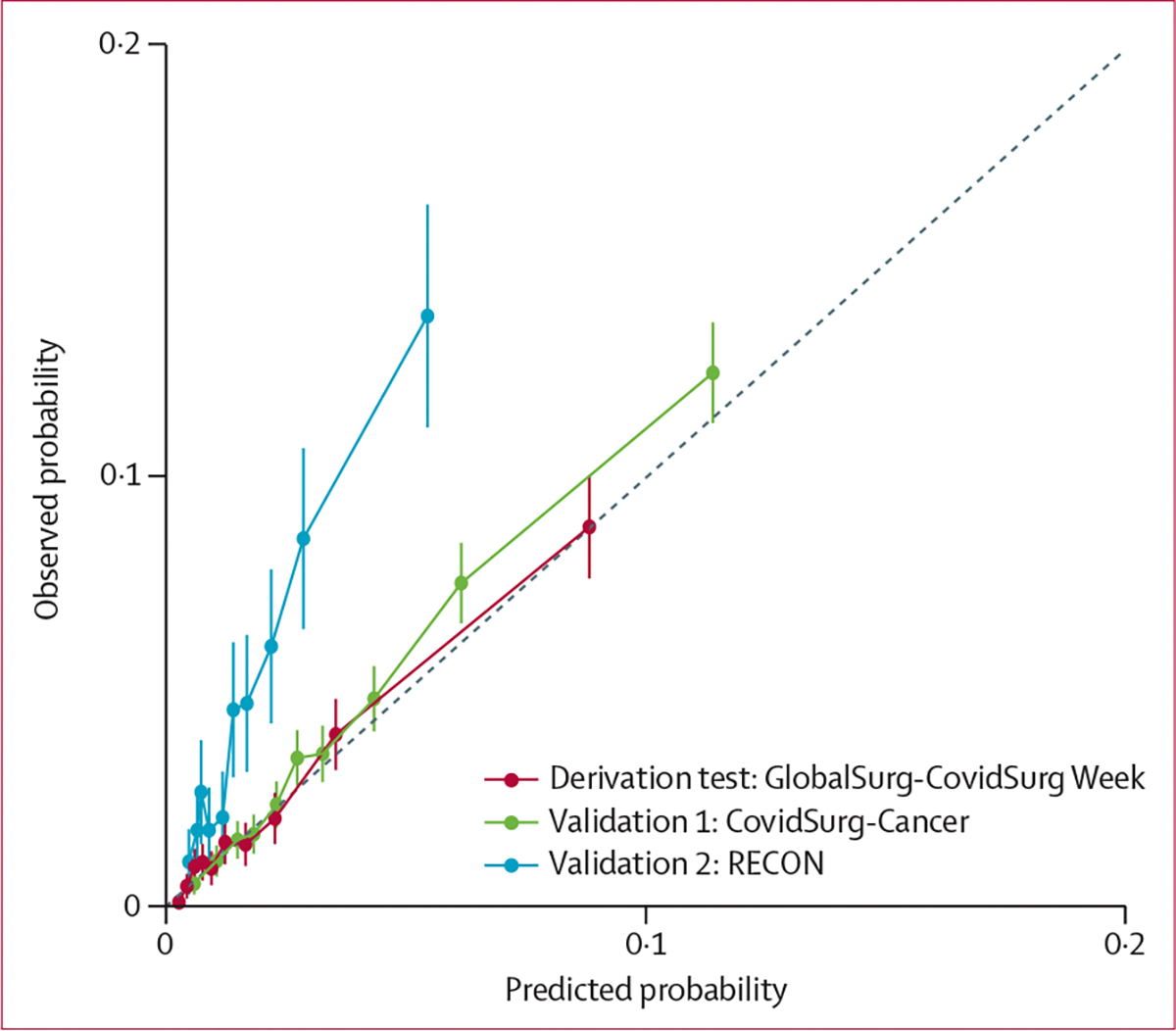
Calibration of GSU-Pulmonary Score

86 231 entered the structured machine learning process, in which patients were split into test and training sets, and the performance of XGBoost and LASSO models was assessed (figure 1). Similar discrimination (appendix p 3) was observed between LASSO (AUROC 0·786, 95% CI 0·774–0·798) and XGBoost (AUROC 0·785, 95% CI 0·772–0·797) models. Variable importance observed in both modelling approaches is summarised in the appendix (p 4). Based on our predefined model selection criteria, given the similar performance, more explainable nature, and increased simplicity of LASSO, we proceeded to develop this model as the GSU-Pulmonary Score. After resampling with 2000 bootstraps in the training set, beta coefficients were averaged and three variables with zero coefficients were dropped. The penalised regression coefficients were then scaled, generating the final pragmatic clinical scoring system, which included ten predictor variables. Results are summarised in table 2. For clinical application, individual point scores for each risk factor level are summed to give an overall point score out of a maximum of 23. The GSU-Pulmonary model was assessed in the derivation (testing) set with AUROC of 0·773 (95% CI 0·751–0·795; figure 2) and good calibration (Brier score 0·020, CITL 0·034, slope 0·954; figure 3).

We estimated a minimum sample size of 193 events and 6403 non-events for external validation based on the incidence of pulmonary complications in the derivation data. In assessment of the adequacy of sample size, both CovidSurg-Cancer (1185 events, 29 303 non-events) and RECON (318 events, 6471 non-events) were sufficient for external validation. Upon external validation, the discrimination of the GSU-Pulmonary Score was moderate in the CovidSurg-Cancer (AUROC 0·746, 95% CI 0·733–0·760) and RECON (AUROC 0·716, 95% CI 0·689–0·744) datasets (figure 2). Calibration again was acceptable in CovidSurg-Cancer (Brier score 0·036, CITL 0·109, slope 1·056), but with some miscalibration in RECON data (Brier score 0·045, CITL 1·040, slope 1·009) showing systematic underestimation of risk (figure 3). Upon subgroup analysis, discrimination remained acceptable across all predefined groups (appendix p 5). In sensitivity analyses to address death as a competing risk, there was little change in performance in CovidSurg-Cancer (AUROC 0·753, 95% CI 0·740–0·767; Brier score 0·036, CITL −0·016, slope 1·041) or RECON (AUROC 0·717, 95% CI 0·688–0·745; Brier score 0·046, CITL 1·059, slope 1·004) validation sets (appendix p 6).

We applied the interpretation framework for external validation studies. In step one (assessing the relatedness of datasets) we identified the following: CovidSurg-Cancer had a similar mix of patient and operations to the development set but included only patients with a malignant indication for surgery; and RECON included patients undergoing major abdominopelvic or thoracic surgery under general anaesthesia in high-income countries, with no (imputed as low) community SARS-CoV-2 risk. Given this restricted population, the spread of the linear predictor (log-odds predictor) was narrower in RECON (appendix pp 7, 9) than in CovidSurg-Cancer (appendix pp 8–9). In step two, we compared the calibration and discrimination of the model across the three datasets (figures 2, 3). In step three, we concluded that CovidSurg-Cancer appeared to evaluate (statistical) reproducibility of the GSU-Pulmonary Score whereas RECON appeared to favour (clinical) transportability.

Prognostic test accuracy metrics across a range of cutoff point scores to rule in or rule out PPC in the whole derivation set (appendix p 12) and cutoffs in validation sets (appendix pp 13–14) are provided. A cut-point score of 5 or less (30 689 [35·6%] of 23 029 patients, PPC rate 0·4%) ruled out PPC with a sensitivity of 93·05%, specificity of 35·82%, positive predictive value of 2·96, and negative predictive value of 99·95. The distribution of point scores and risk estimates is shown in the appendix (p 9). Four risk strata were defined clinically as low risk (0–5 score, PPC rate in whole derivation set 0·45%, n=30 689), intermediate risk (6–10 score, PPC rate 1·68%, n=43 888), high risk (11–15 score, PPC rate 7·37%, n=11 027), and very high risk (≥16 score, PPC rate 14·67%, n=627). Risk groupings across the three datasets are shown in the appendix (p 6). Finally, the GSU-Pulmonary Score calculator was deployed online. The independent PROBAST assessment indicated that the study was at low risk of bias and there were low concerns about applicability (appendix pp 22–30).

Decision curve analysis showed clinical use of the COVIDSurg Pulmonary Score over a range of threshold risks in CovidSurg-Cancer data (appendix p 10); however, our model had limited clinical use at low-risk thresholds in RECON data due to miscalibration and subsequent underestimation of risk. Therefore, we suggest caution when using the model to guide clinical management in patients with a low risk of pulmonary complications in non-COVID-19 data.

## Discussion

This study has defined a novel risk stratification tool (GSU-Pulmonary Score) to estimate the risk of pulmonary complications in adults undergoing elective surgery. It requires simple predictor variables, which are readily available to clinicians across all resource settings and at the point of a decision for surgery. The study was designed and reported according to best-practice guidelines and was at a low risk of bias.^7^ It is the first scoring tool to consider background community SARS-CoV-2 infection rate, allowing dynamic risk prediction in areas with ongoing SARS-CoV-2 transmission and until universal vaccination coverage is achieved.^6^ The score was shown to have acceptable discrimination in two large, well powered, multicountry validation sets, with good calibration in CovidSurg-Cancer and miscalibration in RECON data. Pulmonary events are the major driver of death after elective surgery, and increased in frequency and severity during the COVID-19 pandemic.^4^

We recommend that the GSU-Pulmonary Score be used to inform patient selection, consent, and resource allocation as elective surgery is upscaled during pandemic recovery efforts and beyond.^2,10^ Delays in elective health care represent a major threat to global public health and care for non-communicable disease, particularly for time-critical conditions such as cancer.^33^ As global health-care systems continue to upscale elective surgery to mitigate against further harm for these patients, accurate and accessible risk stratification tools will be essential to inform shared decision making about the timing of surgery. For patients classified at higher risk (including those who remain unvaccinated), vaccination (or a booster dose) can be offered, and surgery might be done under regional or local anaesthetic, or delayed while the patient is preoptimised (eg, preoperative chest physiotherapy, prophylactic mucolytics, or goal-directed haemodynamic therapy) or until community SARS-CoV-2 rates decline.^34^ Critical care providers could use this score to plan bed availability. For patients judged to be at lower risk, it is likely to be safe to continue surgery even when community SARS-CoV-2 rates are high. Decisions to postpone, continue, or cancel surgery are complex, multifactorial, and require deep shared decision making between the patient and the perioperative multidisciplinary team; the likelihood of clinical benefit of surgery would have significant influence on this process. It would therefore be inappropriate to specify exactly how the GSU-Pulmonary Score should be used in decision making. Instead, we present a range of cutoff point scores to rule in or rule out PPC that clinicians can explore during implementation. The incidence of PPC in all three studies was greater than 1·68% (intermediate-risk group cutoff), reflecting the fact that patients undergoing abdominal surgery are at intermediate-to-high risk at baseline. Of note, we did not include patients with recent preoperative SARS-CoV-2 infection in this study and the model is not directly applicable to this group; other data are available to inform clinical decision making here.^8^ Although some of the variability in PPC rates might have been due to inclusion of a small number of patients with undetected SARS-CoV-2, much of this variation would be captured by community SARS-CoV-2 transmission rates; preoperative SARS-CoV-2 testing had little effect on model discrimination and was dropped in penalised regression.

A systematic review and validation study in 2022 identified several other published prognostic models to estimate the risk of pulmonary complications after major abdominal surgery.^6^ In the same RECON cohort study data used in this study, no model showed good discrimination (an AUROC greater than 0·7). Here, in RECON study data, the GSU-Pulmonary Score displayed better discrimination than the best-performing score (Assess Respiratory Risk in Surgical Patients in Catalonia [ARISCAT])^35^ identified in the systematic review (AUROC 0·716, 95% CI 0·689–0·744 *vs* 0·700, 0·683–0·717) and the Score for the Prediction of Postoperative Respiratory Complications ([SPORC-1]; AUROC 0·574, 95% CI 0·556–0·593), although with some miscalibration (CITL close to 1, and predicted risks systemically too low). RECON included major resectional abdominopelvic surgery in high-income countries only. Miscalibration might be due to unmeasured differences in casemix (eg, balance of major and complex-major surgery within the single major surgery predictor variable), differences in practice during the pandemic (eg, proactive risk modification), or system-level factors such as access to care and health behaviours in lower-resource settings (eg, barriers to travel to hospital after discharge, so PPC is less likely to be observed). The RECON dataset was included to improve relevance to countries and regions, which might in the future have very low or no ongoing SARS-CoV-2 transmission.

The GSU-Pulmonary Score has several notable advantages over previous prognostic tools for surgical patients. First, it considers variation in patients’ risk related to community SARS-CoV-2 case notification rates.^8^ Existing COVID-19 risk scores in general medical populations can only stratify risk for patients with active SARS-CoV-2 infection, so are applicable to only a small subset of elective surgery patients and are not designed to reflect the physiological insult of surgery.^7^ Second, it includes only resource-light predictor variables that do not require any additional tests or equipment (in contrast to preoperative oxygen saturation and serum haemoglobin in the ARISCAT score).^35^ It is applicable across resource settings and can be calculated remotely (eg, from electronic health records or during telemedicine consultation). Third, it includes only predictor variables available before the day of surgery, so can support preoperative decision making (in contrast to duration of surgery and emergency procedure in ARISCAT). Fourth, it has been developed and validated in large, international datasets with broad inclusion criteria, making it highly generalisable. Fifth, we did a model comparison study and selected a modelling technique that is readily interpretable by the front-line clinician, improving clinical utility. The finding that advanced structured machine learning approaches are not superior to simple regression, even in very large clinical datasets, has been replicated elsewhere.^33^ We also highlighted that different variables were most important across the two modelling approaches. This has important ethical implications for clinical risk prediction models in the future, whereby high-level decisions (eg, whether a patient should be offered surgery) might be made based on different factors depending on the modelling approach. The interpretation framework for external validation studies suggests that the score is transferable across different perioperative patient groups, including beyond the COVID-19 pandemic.

There are also several limitations to this analysis. First, some clinically relevant predictor variables were not included in score development (eg, cardiac history, preoperative physiology, and cardiopulmonary exercise testing). This was a pragmatic decision to facilitate high data quality across large, prospective studies, and to ensure that the resulting model was applicable to low-resource settings. The output estimates should be implemented alongside holistic assessment of the patient’s status and with shared decision making. Data on ethnicity were also not collected due to lack of a global, culturally sensitive classification system. Improving reporting of ethnicity in model development is an important emerging area of research. Second, all clinical predictor variables were collected categorically. This simplification might have reduced statistical power,^36^ and might explain a degree of miscalibration in RECON data. Third, data from the derivation and validation (CovidSurg-Cancer) sets came from the COVID-19 pandemic period. Although there continue to be areas of the world with high rates of SARS-CoV-2 infection, the individual risk of pulmonary complications in infected surgical patients is likely to be lower in the omicron-variant era and in vaccinated patients. Despite this, preliminary reports from the CovidSurg-3 study (NCT05161299; prospective data collection between December, 2021, and February, 2022) indicate that high-risk patients (eg, those aged >70 years, ASA grade 3–5) remain at very high risk of pulmonary complications and death with perioperative SARS-CoV-2 infection.^37^ To explore the implications of this on model performance, we also undertook model validation in a non-COVID dataset (RECON). Model re-calibration might be required in the future as the community gains a deeper understanding of the contemporary phenotype of COVID-19 in a surgical population. The directionality of effect for high versus low SARS-CoV-2 risk in upper-middle-income countries was reversed in comparison with other income groups; this might reflect within-group variation in access to testing and reporting rather than a true biological effect. Fourth, we dealt with missing data (<0·1% overall) using complete-case analysis. However, several clinical and simulation studies have shown little effect of multiple imputation methods where there are very low rates of missing data.^20^ Fifth, it was not possible to mask front-line clinical staff who recorded outcome assessment to several predictor variables, as these were readily apparent. However, the primary outcome measure required clinical imaging studies (chest x-ray or CT) for diagnosis that were reported by independent radiologists, minimising risk of bias. We adopted a composite outcome measure, and the model might not discriminate equally for each included endpoint. Sixth, the score cannot be applied to patients undergoing procedures under deep sedation, who are at risk of pulmonary complications. Seventh, death presented a competing risk for pulmonary complications and shared a common casual pathway for some patients. We showed robust performance of the score in a sensitivity analysis, but were unable to account for this during model derivation. Eighth, more models and preprocessing could have been implemented, including using methods for managing class imbalance (ie, oversampling or under-sampling, or Synthetic Minority Over-Sampling Technique). However, these are criticised in clinical datasets due to risk of miscalibration (strong overestimation of probability to belong to the minority class), and we opted not to adopt these here.^38^ Ninth, we adopted two techniques for model com parison but did not include other interpretable model types that can account for non-linear covariables, such as generalised additive modelling. This could be an important consideration in future work. Tenth, only one of three cohort studies (RECON) explicitly preplanned the development and validation of a prognostic model in its study protocol. Finally, we have not been able to compare performance of the GSU-Pulmonary Score with other models in a dataset, which includes all operation types and indications; our inferences about superior performance of the model relate to abdominal surgery only, and we have not compared it with SPORC-2, which was published after study completion. This highlights an area for future comparisons.

## Supplementary Material

supplementary

## Figures and Tables

**Table 1:** Comparison of derivation and validation datasets

	Derivation: GlobalSurg-CovidSurg Week (n=86 231)	Validation: CovidSurg-Cancer (n=30 492)	Validation: RECON (n=6789)

**Patient factors**			
Age, years			
18–29	9106 (10·6%)	636 (2·1%)	276 (4·1%)
30–39	12 176 (14·1%)	1708 (5·6%)	555 (8·2%)
40–49	13 272 (15·4%)	3745 (12·3%)	983 (14·5%)
50–59	15 703 (18·2%)	6697 (22·0%)	1466 (21·6%)
60–69	16 665 (19·3%)	8347 (27·4%)	1623 (23·9%)
70–79	13 742 (15·9%)	6954 (22·8%)	1390 (20·5%)
80–89	5110 (5·9%)	2265 (7·4%)	484 (7·1%)
>90	457 (0·5%)	140 (0·5%)	12 (0·2%)
Sex			
Female	47 219 (54·8%)	18 329 (60·1%)	3905 (57·5%)
Male	39 012 (45·2%)	12 163 (39·9%)	2884 (42·5%)
ASA grade			
Grade 1	21 667 (25·1%)	6431 (21·1%)	1048 (15·4%)
Grade 2	43 099 (50·0%)	15 752 (51·7%)	3903 (57·5%)
Grade 3	19 057 (22·1%)	7912 (26·0%)	1733 (25·5%)
Grade 4 and 5	2408 (2·8%)	397 (1·3%)	105 (1·5%)
Revised Cardiac Risk Index			
0	38 541 (44·7%)	9245 (30·3%)	4112 (60·6%)
1	37 688 (43·7%)	15 896 (52·1%)	2210 (32·6%)
2	7595 (8·8%)	4284 (14·1%)	448 (6·6%)
≥3	2407 (2·8%)	1067 (3·5%)	19 (0·3%)
Pre-existing respiratory disease			
No	77 747 (90·2%)	27 282 (89·5%)	5611 (82·6%)
Yes	8484 (9·8%)	3210 (10·5%)	1178 (17·4%)
Current smoker			
No	72 830 (84·5%)	27 311 (89·6%)	5852 (86·2%)
Yes	13 401 (15·5%)	3181 (10·4%)	937 (13·8%)
**Disease factors**			
Compartment			
Abdominopelvic	43 761 (50·7%)	16 817 (55·2%)	6789 (100·0%)
Head and neck	8228 (9·5%)	3823 (12·5%)	NA
Limb	12 514 (14·5%)	196 (0·6%)	NA
Other	17 516 (20·3%)	7226 (23·7%)	NA
Thoracic	4212 (4·9%)	2430 (8·0%)	NA
Indication			
Benign	64 822 (75·2%)	NA	3884 (57·2%)
Cancer	21 409 (24·8%)	30 492 (100·0%)*	2905 (42·8%)
Preoperative SARS-CoV-2 test			
Negative	61 339 (71·1%)	14 128 (46·3%)	6789 (100·0%)†
Not performed	24 892 (28·9%)	16 364 (53·7%)	NA
**Operation factors**			
Anaesthesia type			
General	63 235 (73·3%)	28 771 (94·4%)	6789 (100·0%)
Regional or local	22 996 (26·7%)	1721 (5·6%)	NA
Operation grade			
Major	53 061 (61·5%)	24 439 (80·1%)	6789 (100·0%)
Minor	33 170 (38·5%)	6053 (19·9%)	NA
**Location factors**			
Hospital type			
COVID-19-free surgical pathway	71 809 (83·3%)	8097 (26·6%)	6789 (100·0%)
Hospital with no defined pathway	14 422 (16·7%)	22 395 (73·4%)	NA
Country income			
High income	59 284 (68·8%)	24 365 (79·9%)	6789 (100·0%)
Upper-middle income	14 614 (16·9%)	3385 (11·1%)	NA
Low-middle income	12 333 (14·3%)	2742 (9·0%)	NA
Community SARS-CoV-2 risk			
High	57 229 (66·4%)	16 770 (55·0%)	NA
Low	29 002 (33·6%)	13 722 (45·0%)	6789 (100·0%)†
Country income × SARS-CoV-2 risk			
High income, high SARS-CoV-2 risk	48 375 (56·1%)	14 814 (48·6%)	NA
High income, low SARS-CoV-2 risk	10 909 (12·7%)	9551 (31·3%)	6789 (100·0%)†
Upper-middle income, high SARS-CoV-2 risk	8339 (9·7%)	1309 (4·3%)	NA
Upper-middle income, low SARS-CoV-2 risk	6275 (7·3%)	2076 (6·8%)	NA
Low-middle income, high SARS-CoV-2 risk	515 (0·6%)	647 (2·1%)	NA
Low-middle income, low SARS-CoV-2 risk	11 818 (13·7%)	2095 (6·9%)	NA
**Outcome measures**			
Postoperative pulmonary complication			
Negative	84 450 (97·9%)	29 307 (96·1%)	6471 (95·3%)
Positive	1781 (2·1%)	1185 (3·9%)	318 (4·7%)
Death			
Yes	601 (0·7%)	320 (1·05%)	27 (0·4%)
No	85 630 (99·3%)	30 172 (99·0%)	6566 (99·6%)
Missing	3	24	223

ASA=America Society of Anesthesiologists. NA=not applicable.

* Includes cancer indication only.

† Imputed as the null value as data were collected before the COVID-19 pandemic.

**Table 2:** Final GSU-Pulmonary Score model description (derivation dataset)

	No postoperative pulmonary complications	Postoperative pulmonary complications	Odds ratio (univariable)	Mean coefficients (LASSO)	Odds ratio (LASSO)	Point score

**Age, years**						
18–29	6751 (10·7)	66 (5·0)	1·01 (0·73 to 1·40)	0·019 (0·012 to 0·025)	1·019	0
30–39	9030 (14·3)	87 (6·6)	··	··	··	0
40–49	9822 (15·5)	154 (11·6)	1·63 (1·25 to 2·13)	0·176 (0·17 to 0·182)	1·192	1
50–59	11 571 (18·3)	227 (17·1)	2·04 (1·59 to 2·62)	0·158 (0·153 to 0·163)	1·171	0
60–69	12 122 (19·1)	335 (25·3)	2·87 (2·27 to 3·66)	0·299 (0·293 to 0·304)	1·348	1
70–79	10 035 (15·8)	309 (23·3)	3·20 (2·53 to 4·08)	0·382 (0·376 to 0·388)	1·465	1
80–89	3686 (5·8)	133 (10·0)	3·75 (2·86 to 4·93)	0·594 (0·587 to 0·601)	1·811	2
≥90	331 (0·5)	14 (1·1)	4·39 (2·37 to 7·55)	0·882 (0·867 to 0·897)	2·416	3
**Sex**						
Female	34 908 (55·1)	535 (40·4)	··	··	··	0
Male	28 440 (44·9)	790 (59·6)	1·81 (1·62 to 2·03)	0·393 (0·39 to 0·395)	1·481	1
**ASA grade**						
1	16 031 (25·3)	143 (10·8)	··	··	··	0
2	31 963 (50·5)	443 (33·4)	1·55 (1·29 to 1·88)	0·086 (0·082 to 0·09)	1·09	0
3	13 712 (21·6)	570 (43·0)	4·66 (3·89 to 5·62)	0·715 (0·71 to 0·719)	2·044	2
4 or 5	1642 (2·6)	169 (12·8)	11·54 (9·19 to 14·51)	1·138 (1·131 to 1·144)	3·12	3
**Revised Cardiac Risk Index**						
0	28 632 (45·2)	263 (19·8)	··	··	··	0
1	27 661 (43·7)	610 (46·0)	2·40 (2·08 to 2·78)	0·311 (0·307 to 0·315)	1·365	1
2	5415 (8·5)	303 (22·9)	6·09 (5·15 to 7·21)	0·566 (0·561 to 0·571)	1·761	2
≥3	1640 (2·6)	149 (11·2)	9·89 (8·03 to 12·15)	0·769 (0·763 to 0·775)	2·158	2
**Pre-existing respiratory disease**						
No	57 217 (90·3)	1052 (79·4)	··	··	··	0
Yes	6131 (9·7)	273 (20·6)	2·42 (2·11 to 2·77)	0·483 (0·48 to 0·486)	1·621	1
**Current smoker** *						
No	53 592 (84·6)	1071 (80·8)	··	··	··	0
Yes	9756 (15·4)	254 (19·2)	1·30 (1·13 to 1·49)	0·125 (0·122 to 0·129)	1·134	0
**Compartment**						
Abdominopelvic	32 281 (51·0)	649 (49·0)	··	··	··	0
Head and neck	6043 (9·5)	110 (8·3)	0·91 (0·73 to 1·11)	0·073 (0·068 to 0·078)	1·076	0
Limb	9208 (14·5)	111 (8·4)	0·60 (0·49 to 0·73)	0·054 (0·049 to 0·059)	1·056	0
Other	13 010 (20·5)	141 (10·6)	0·54 (0·45 to 0·65)	−0·173 (−0·178 to −0·168)	0·841	−1
Thoracic	2806 (4·4)	314 (23·7)	5·57 (4·83 to 6·40)	0·904 (0·901 to 0·908)	2·471	3
**indication**						
Benign	47 878 (75·6)	755 (57·0)	··	··	··	0
Cancer	15 470 (24·4)	570 (43·0)	2·34 (2·09 to 2·61)	0·523 (0·521 to 0·526)	1·688	2
**Preoperative SARS-CoV-2 test** *						
Negative	45 047 (71·1)	960 (72·5)	··	··	··	0
Not performed	18 301 (28·9)	365 (27·5)	0·94 (0·83 to 1·06)	0·164 (0·161 to 0·167)	1·179	0
**Anaesthesia type**						
Regional or local	17067 (26·9)	138 (10·4)	··	··	··	0
General	46281 (73·1)	1187 (89·6)	3·17 (2·67 to 3·80)	0·576 (0·572 to 0·58)	1·779	2
**Operation grade**						
Minor	24 750 (39·1)	197 (14·9)	··	··	··	0
Major	38 598 (60·9)	1128 (85·1)	3·67 (3·16 to 4·29)	0·744 (0·74 to 0·747)	2·104	2
**Hospital type** *						
COVID-19-free surgical pathway	52 788 (83·3)	1077 (81·3)	··	··	··	0
Hospital with no defined pathway	10 560 (16·7)	248 (18·7)	1·15 (1·00 to 1·32)	−0·03 (−0·034 to −0·027)	0·97	0
**Country income × SARS-CoV-2 risk**						
High income, high SARS-CoV-2 risk	35 563 (56·1)	705 (53·2)	1·30 (1·07 to 1·58)	0·289 (0·284 to 0·293)	1·334	1
High income, low SARS-CoV-2 risk	8055 (12·7)	123 (9·3)	··	··	··	0
Upper-middle income, high SARS-CoV-2 risk	6121 (9·7)	142 (10·7)	1·52 (1·19 to 1·94)	0·758 (0·753 to 0·764)	2·134	2
Upper-middle income, low SARS-CoV-2 risk	4555 (7·2)	154 (11·6)	2·21 (1·74 to 2·82)	1·039 (1·033 to 1·044)	2·825	3
Low-middle income, high SARS-CoV-2 risk	379 (0·6)	18 (1·4)	3·11 (1·82 to 5·02)	1·22 (1·208 to 1·232)	3·387	4
Low-middle income, low SARS-CoV-2 risk	8675 (13·7)	183 (13·8)	1·38 (1·10 to 1·74)	0·851 (0·846 to 0·857)	2·343	3
Intercept	··	··	··	−6·836 (−6·844 to −6·827)	−21	··

Model description after 2000 bootstraps and averaged coefficients. To generate the final scaled calculator score, mean coefficients were multiplied by 3 and rounded. All categorical variables were dummified (ie, each different category acts as an independent variable). Where multiple factor levels (eg, age group) score 0 points, this reflects non-linear patterns of risk over different groups. During penalised regression using LASSO, the coefficients for all factor levels have shrunk towards 0 for some covariables. This indicates that they had provided little discriminative ability and therefore were not selected for inclusion in the final model (eg, current smoker). ASA=America Society of Anesthesiologists. LASSO=least absolute shrinkage and selection operator.

* Covariables that were removed from the final model after penalised regression.

## Data Availability

Full, anonymised data are available upon request to the study management groups and successful completion of a data sharing agreement.

## Research in context

### Evidence before this study

The global backlog of elective surgery is now a major public health priority, but patients remain at risk of postoperative pulmonary complications, which are the most common cause of death after surgery. As elective capacity is upscaled to address waiting lists, individual risk scoring is needed to inform patient consent, case prioritisation, and resource allocation during the COVID-19 recovery period. We previously completed a systematic review of articles reporting risk prediction models for postoperative pulmonary complications. We searched MEDLINE and Embase from database inception to March 1, 2020 using the search terms “surgery”, “pulmonary complication”, and “postoperative pulmonary complication”; of the 29 models identified, we validated six in an external cohort. The findings were published in 2022 in *The Lancet Digital Health*. We then updated this search to include studies up to Nov 8, 2023. We identified 33 unique prognostic models. None of the six models that underwent external validation showed good discrimination (defined as the area under the receiver operating curve [AUROC] lower confidence interval estimate of ≥0·70) in identifying postoperative pulmonary complications. All six were at high or unclear risk of bias on PROBAST assessment. Existing risk scores did not evaluate structured machine learning methods, and often included intraoperative variables that were either not available at the time of the decision to operate, or were inaccessible in resource-constrained health systems.

### Added value of this study

The GSU-Pulmonary Score is a novel prognostic risk score that aims to estimate the risk of postoperative pulmonary complications in adults undergoing elective surgery, based on ten preoperative variables routinely accessible in preoperative settings. To our knowledge, this is the first score to consider background community SARS-CoV-2 infection rate, allowing dynamic risk prediction in areas with ongoing SARS-CoV-2 transmission. The model was derived from a prospective international dataset of 86 231 patients from a diverse range of hospitals and countries, and discrimination and calibration were explored in two external prospective datasets of 37 281 patients in total. The discrimination of the model was superior to that of the best-performing score (ARISCAT) in our 2022 systematic review (AUROC 0·716 *vs* 0·700), with the caveat that ARISCAT included abdominal, abdominothoracic, and pelvic operations only.

### Implications of all the available evidence

The GSU-Pulmonary Score includes simple, preoperative variables that can be deployed by a patient’s bedside or in an outpatient clinic in any country and setting, and considers baseline community SARS-CoV-2 infection rates. It can be implemented in individual risk assessment for patient prioritisation and resource management during the ongoing COVID-19 recovery period. It will support front-line clinicians and policy makers to safely scale capacity for elective surgery to address growing waiting lists around the world. In case of a future emerging threat from a high-risk respiratory virus, we anticipate that the model could be recalibrated to address changing risks of pulmonary complications, acting as a sleeping study to improve pandemic preparedness.

## NIHR Global Health Research Unit on Global Surgery

*Writing group*: Laura Bravo, Joana F F Simões, Victor R Cardoso, Adewale Adisa, Maria L A Aguilera, Alexis Arnaud, Bruce Biccard, Jose Calvache, Saisakul Chernbumroong, Muhammed Elhadi, Dhruv Ghosh, Rohan Gujjuri, Ewen Harrison, Michael W S Ho, Veerappan Kasivisvanathan, Omar Kouli, Hans Lederhuber, Elizabeth Li, Markus W Löffler, Arda Isik, Hani Marcus, Janet Martin, Kenneth A McLean, Ana Minaya-Bravo, María Marta Modolo, Dmitri Nepogodiev, Gianluca Pellino, Maria Picciochi, Peter Pockney, Gabriëlle van Ramshorst, Aya Riad, Raza Sayyed, Malin Sund, Georgios Gkoutos, Aneel A Bhangu, James C Glasbey. All other members are listed in the appendix.
